# miR-23b-3p regulates the chemoresistance of gastric cancer cells by targeting ATG12 and HMGB2

**DOI:** 10.1038/cddis.2015.123

**Published:** 2015-05-21

**Authors:** Y An, Z Zhang, Y Shang, X Jiang, J Dong, P Yu, Y Nie, Q Zhao

**Affiliations:** 1 State Key Laboratory of Cancer Biology and Xijing Hospital of Digestive Diseases, The Fourth Military Medical University, 127 Changle Western Road, Xi'an, Shaanxi 710032, China; 2 Department of General Surgery, General Hospital of Jinan Military Command, Jinan, China; 3 Department of Biochemistry and Molecular Biology, Zhejiang Provincial Key Laboratory of Pathophysiology, Ningbo University School of Medicine, Ningbo, Zhejiang 315211, China

## Abstract

Chemotherapy is an important treatment modality for gastric cancer (GC); however, it usually fails because of drug resistance, especially multidrug resistance (MDR). Previously, we found a novel subset of MDR-associated microRNAs (miRNAs) through high-throughput functional screening. In this report, we investigated the exact roles and mechanisms of miR-23b-3p in the MDR of GC. Using gain or loss-of-function in *in vitro* and *in vivo* experiments, we found that overexpression of miR-23b-3p reversed cancer cell resistance to multiple chemotherapeutics *in vitro* and sensitize tumors to chemotherapy *in vivo*. Reporter gene assay and western blot analysis showed that ATG12 and HMGB2 were the direct targets of miR-23b-3p. Meanwhile, ATG12 and HMGB2 were positively associated with the occurrence of autophagy. Reducing the expression of these target genes by siRNA or inhibition of autophagy both sensitized GC cells to chemotherapy. These findings suggest that a miR-23b-3p/ATG12/HMGB2/autophagy-regulatory loop has a critical role in MDR in GC. In addition, miR-23b-3p could be used as a prognostic factor for overall survival in GC. In conclusion, our data demonstrated that miR-23b-3p inhibited autophagy mediated by ATG12 and HMGB2 and sensitized GC cells to chemotherapy, and suggested the potential application of miR-23b-3p in drug resistance prediction and treatment.

Gastric cancer (GC) has been the second leading cause of cancer-related deaths worldwide over the past century.^1, 2^ Because of the lack of effective techniques for early diagnosis, most GC patients are diagnosed at an advanced stage of the disease. For these patients, chemotherapy is the first-line treatment. However, even though many novel chemotherapeutic drugs are used in clinical practice, chemotherapeutic approaches fail because of intrinsic or acquired drug resistance, particularly multidrug resistance (MDR).^3, 4^ The mechanisms underlying MDR have been widely studied and include redistribution of the intracellular accumulation of drugs, alteration of drug targets, enhanced DNA repair activity and inactivation of apoptosis pathways.^5, 6, 7, 8^ Although these MDR mechanisms have been extensively explored, the key determinants of this phenomenon remain largely unclear.

MicroRNAs (miRNAs) are a class of noncoding RNAs of ~21–25 nucleotides that negatively regulate gene expression at the post-transcriptional level by targeting the 3′-untranslated regions (3′-UTRs) of specific mRNAs via base pairing, thereby inducing mRNA degradation or translational repression.^9^ Accumulating evidence has shown that miRNAs have an important role in the MDR of various types of cancer, including GC.^10, 11, 12^ In particular, miR-200c, miR-15b, miR-16 and miR-508-5p^13, 14, 15^ have been reported to be involved in the MDR of GC; however, there is still a lack of available data regarding the potential role of miRNAs in the chemoresistance of GC.

In a previous study, using an miRNA array and a functional screening strategy, we found that miR-23b-3p had a potential role in reversing drug resistance in GC.^14, 15^ As a member of miR-23b/27b/24-1 cluster, dysregulation of miR-23b-3p has been reported in many types of cancer. miR-23b-3p was downregulated in human colon cancer, which regulates FZD7 or MAP3k1 to mediate the multiple steps of metastasis *in vivo.*^16^ In addition, miR-23b-3p as an oncogenic miRNA inhibited PTEN in renal cell carcinoma.^17^ Meanwhile, expression of miR-23b-3p was upregulated in radiation-induced thymic lymphoma.^18^ Interestingly, reduced levels of miR-23b-3p increased levels of autophagy to promote radioresistance in pancreatic cancer cells.^19^ However, the association between miR-23b-3p and GC has not been previously reported. In this report, we show that miR-23b-3p can sensitize GC cells to chemotherapy. In addition, we identify autophagy-related gene 12 (ATG12) and high-mobility group box 2 (HMGB2) as two direct and functional targets of miR-23b-3p; these targets are overexpressed in GC MDR cells and show a positive correlation with both autophagy and chemoresistance.

## Results

### miR-23b-3p regulates the sensitivity of GC cells to chemotherapeutic agents *in vitro*

**Figure 1:**
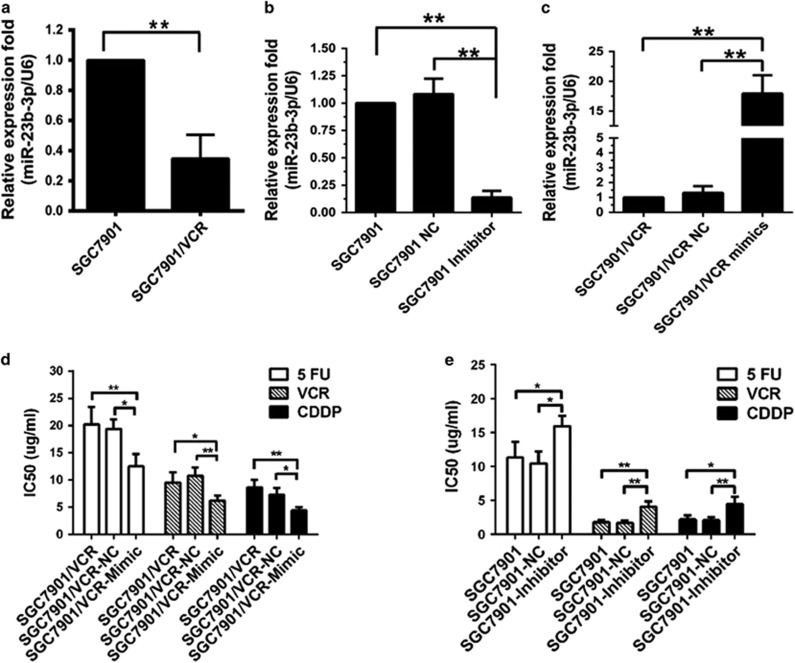
Expression and function of miR-23b-3p in GC cells. (**a**) Downregulation of miR-23b-3p was verified by qRT-PCR in SGC7901/VCR cells. (**b** and **c**) Expression of miR-23b-3p was studied after transfection of GC cells with inhibitors, mimics or NC. (**d** and **e**) miR-23b-3p negatively regulated the drug sensitivity of GC cells to chemotherapeutic agents *in vitro*. *P<0.05, **P<0.01

To verify the results of our previous work,^14, 15^ we first performed qRT-PCR analysis of miR-23b-3p expression in SGC7901 and SGC7901/VCR (vincristine) cells. qRT-PCR showed that compared with SGC7901 cells, miR-23b-3p was significantly downregulated in SGC7901/VCR cells (Figure 1a). In addition, SGC7901 and SGC7901/VCR cells were transiently transfected with miR-23b-3p inhibitors (100 nM) or mimics (50 nM; Figures 1b and c), and 3-(4,5-di-methyl-2-thiazolyl)-2,5-diphenyl-2H tetrazolium bromide (MTT) assays were performed to determine cell growth curve (Supplementary Figure 1) and 50% inhibition of growth (IC_50_) values. Overexpression of miR-23b-3p dramatically enhanced the sensitivity of SGC7901/VCR cells to 5-fluorouracil (5-FU), VCR and CDDP (Figure 1d). Transfection of SGC7901 cells with a specific inhibitor of miR-23b-3p increased the IC_50_ values for these three chemotherapeutic agents (Figure 1e).

### ATG12 and HMGB2 are direct targets of miR-23b-3p

**Figure 2:**
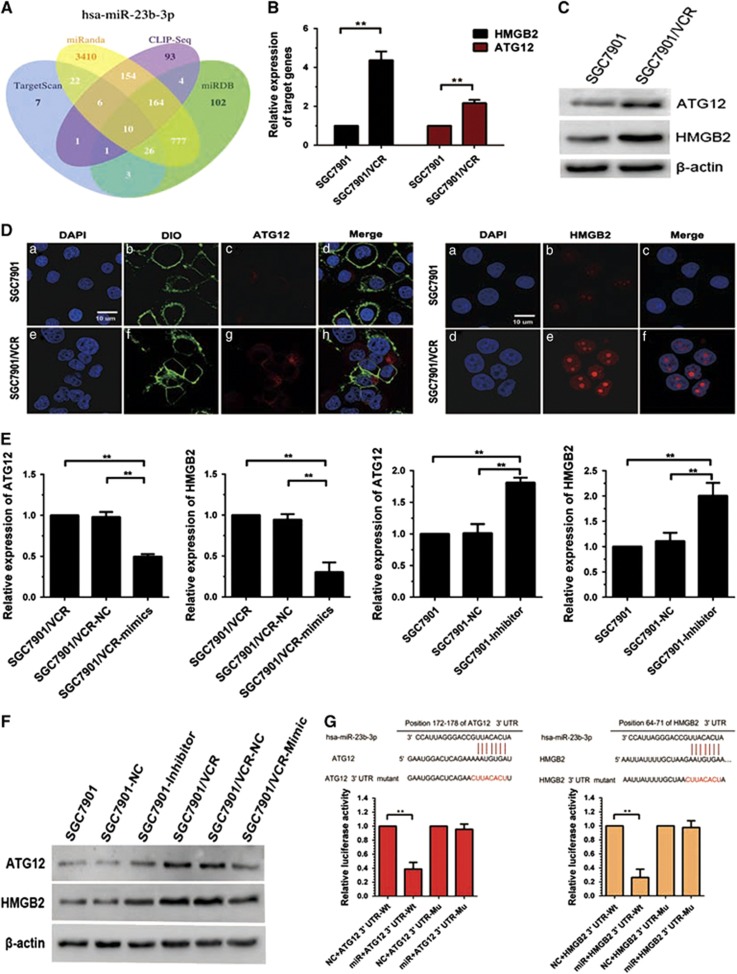
ATG12 and HMGB2 are direct target genes of miR-23b-3p. (**A**) Bioinformatic analysis predicted the target genes of miR-23b-3p. (**B**–**D**) ATG12 and HMGB2 were evaluated using qRT-PCR, western blotting and CLSM (blue fluorescence indicates nuclei stained with DAPI, green indicates cytoplasmic membrane stained with DIO and red indicates the proteins expressed by the target genes) in SGC7901/VCR cells and SGC7901 cells. (**E** and **F**) GC cells were transfected with miR-23b-3p mimics, inhibitor or NC, and the expression of ATG12 and HMGB2 was determined using qRT-PCR and western blot analysis. (**G**) Dual luciferase assays were performed in SGC7901/VCR cells after cotransfection with wild-type or mutant ATG12 and HMGB2 3'-UTR plasmids and NC or miR-23b-3p mimics. *P<0.05, **P<0.01

**Figure 3:**
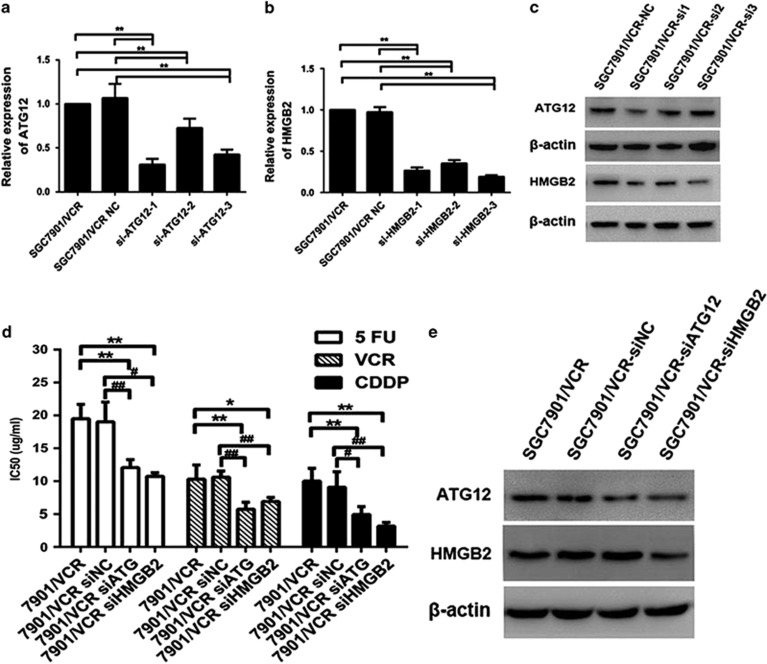
Reduced expression of ATG12 and HMGB2 increases the sensitivity of SGC7901/VCR cells to chemotherapeutic agents. (**a**–**c**) Expression levels of ATG12 and HMGB2 in SGC7901/VCR cells were significantly reduced by siRNAs. (**d**) Silencing of ATG12 or HMGB2 sensitized SGC7901/VCR cells to chemotherapeutic agents and decreased IC50 values. (**e**) ATG12 levels were decreased when HMGB2 was downregulated by siRNA. *P<0.05, **P<0.01

To predict the target genes of miR-23b-3p, we used the bioinformatics algorithms TargetScan (http://www.targetscan.org), miRanda (http://www.microrna.org/microrna/home.do), CLIP-seq (http://en.wikipedia.org/wiki/CLIP#miRNA_target_detection) and miRDB (http://mirdb.org; Figure 2A). ATG12 and HMGB2 were selected as potential target genes of miR-23b-3p. qRT-PCR, western blot and confocal laser scanning microscopy (CLSM) indicated that ATG12 and HMGB2 were upregulated in SGC7901/VCR cells compared with SGC7901 cells (Figures 2B–D). In addition, restoration of miR-23b-3p was sufficient to inhibit ATG12 and HMGB2 expression at both the mRNA and protein levels in SGC7901/VCR cells, whereas downregulation of miR-23b-3p led to the opposite changes in ATG12 and HMGB2 (Figures 2E and F). A dual-luciferase reporter system was used to determine whether ATG12 and HMGB2 were direct targets of miR-23b-3p. Overexpression of miR-23b-3p significantly suppressed the firefly luciferase reporter activity of the wild-type ATG12 and HMGB2 3′-UTR, but did not affect the mutant 3′-UTR in 7901/VCR cells (Figure 2G). Taken together, these results demonstrate that ATG12 and HMGB2 are the direct targets of miR-23b-3p. To further explore the function of ATG12 and HMGB2 in the MDR of GC, three pairs of small interfering RNAs (siRNAs) targeting ATG12 and HMGB2 were synthesized (Supplementary Tables 1 and 2), and their inhibitory effects were confirmed using qRT-PCR and western blotting (Figures 3a–c). Remarkably, silencing of either ATG12 or HMGB2 sensitized SGC7901/VCR cells to chemotherapeutic agents and decreased the IC_50_ values for these drugs (Figure 3d). Interestingly, we found that the silencing of HMGB2 reduced the expression level of ATG12, whereas the expression of HMGB2 was not markedly altered by a siRNA targeting ATG12 (Figure 3e). These data suggest that ATG12 and HMGB2, both of which have important roles in MDR of GC, are direct functional target genes of miR-23b-3p.

### Chemoresistant GC cells exhibit increased autophagy

**Figure 4:**
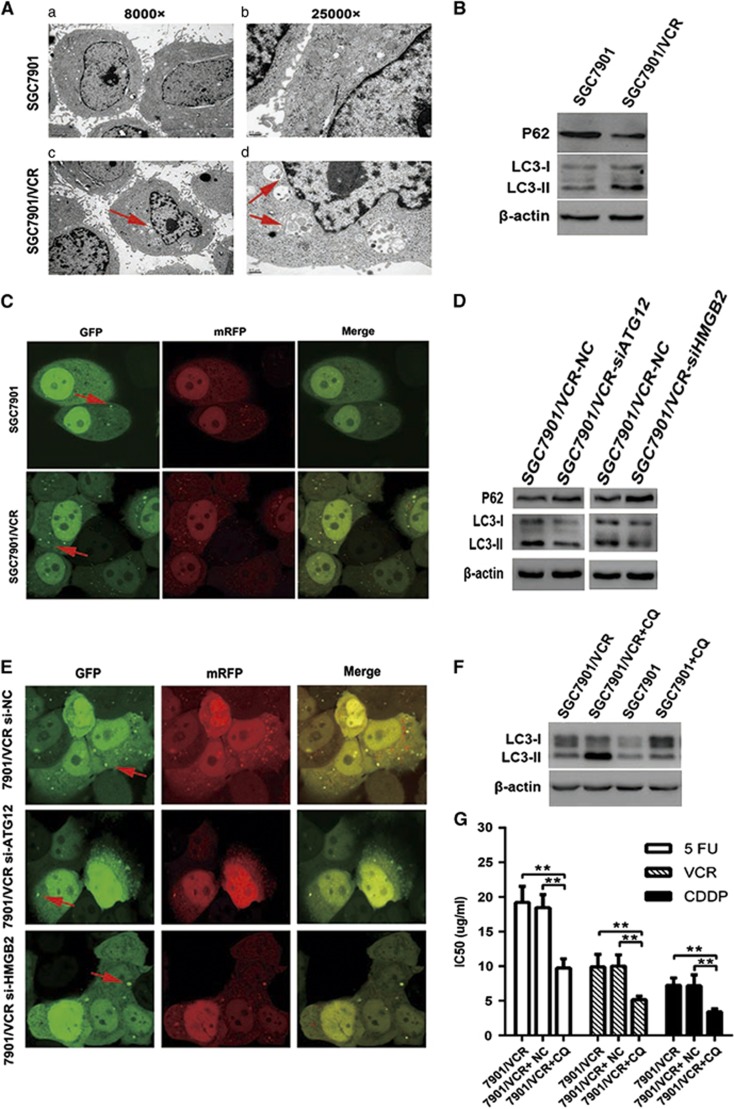
Chemoresistant cells exhibit increased autophagy and inhibition of autophagy restores the sensitivity of chemoresistant cells to chemotherapy. (**A**) Autophagy was evaluated in SGC7901 and SGC7901/VCR cells using TEM. (**B**) Western blot analysis was used to evaluate the expression level of LC3 and p62. (**C**) GC cells that stably express the mRFP-GFP-LC3 fusion protein were established and observed under CLSM. (**D** and **E**) Western blotting and CLSM were used to evaluate the effect of siRNA against ATG12 or HMGB2 on autophagy in SGC7901/VCR cells. (**F**) SGC7901/VCR and SGC7901 cells were not treated or treated with 20 *μ*mol/l CQ for 24 h before being subjected to western blot analysis for LC3 expression. (**G**) CQ greatly enhanced the sensitivity of SGC7901/VCR cells to chemotherapeutic agents; however, this effect was not noticeable in SGC7901 cells *P<0.05, **P<0.01. The arrows indicate autophagosomes (**A**, **C** and **E**)

ATG12 is an important protein in autophagy. HMGB2 is a member of the HMG box family and acts as a damage-associated molecular pattern molecule.^20^ Therefore, we hypothesized that SGC7901/VCR cells may exhibit increased autophagy. Autophagosomes were evaluated in this cell model using transmission electron microscopy (TEM), CLSM and western blotting. TEM revealed a marked accumulation of autophagosomes in the cytoplasm of SGC7901/VCR cells compared with SGC7901 cells (Figure 4A). We then analyzed the processing of LC3-I protein to LC3-II, which is a hallmark of autophagy, using western blot analysis. Increased LC3-II expression and an accompanying decrease in p62 expression were clearly detected in SGC7901/VCR cells compared with SGC7901 cells (Figure 4B). p62 was also used as a marker of autophagy. As p62 accumulates when autophagy is inhibited, and decreased levels can be observed when autophagy is induced. Therefore, p62 was downregulated, also indicating increased autophagy in SGC7901/VCR cells. To confirm these results, we then established a GC cell model that stably expresses an mRFP-GFP-LC3 fusion protein. SGC7901/VCR cells showed a higher mRFP-GFP-LC3 signal than parental SGC7901 cells, indicating that autophagy is enhanced when GC cells develop chemoresistance (Figure 4C). Taken together, these results suggest that chemoresistant GC cells exhibit increased autophagy.

### Inhibition of autophagy restores the chemosensitivity of chemoresistant cells

It was confirmed that silencing of ATG12 or HMGB2 increased the sensitivity of SGC7901/VCR to drugs (Figure 3d). The next question was whether ATG12 or HMGB2 siRNAs could regulate autophagy. Western blot analysis and CLSM indicated that autophagy was markedly decreased in SGC7901/VCR cells that were transfected with siRNAs targeting ATG12 or HMGB2 (Figures 4D and E). In addition, to determine whether autophagy truly contributes to chemoresistance in GC cells, we treated the cells with chloroquine (CQ). As shown in Figure 4F, increased expression of LC3II was observed in SGC7901/VCR cells after 24 h of treatment with CQ. In addition, MTT assays showed that CQ greatly enhanced the sensitivity of SGC7901/VCR cells to chemotherapeutic agents (Figure 4G). These data show that CQ and siRNAs targeting ATG12 and HMGB2 inhibited autophagic activity and sensitized chemoresistant GC cells to chemotherapy.

### miR-23b-3p inhibits autophagy by regulating ATG12 and HMGB2

**Figure 5:**
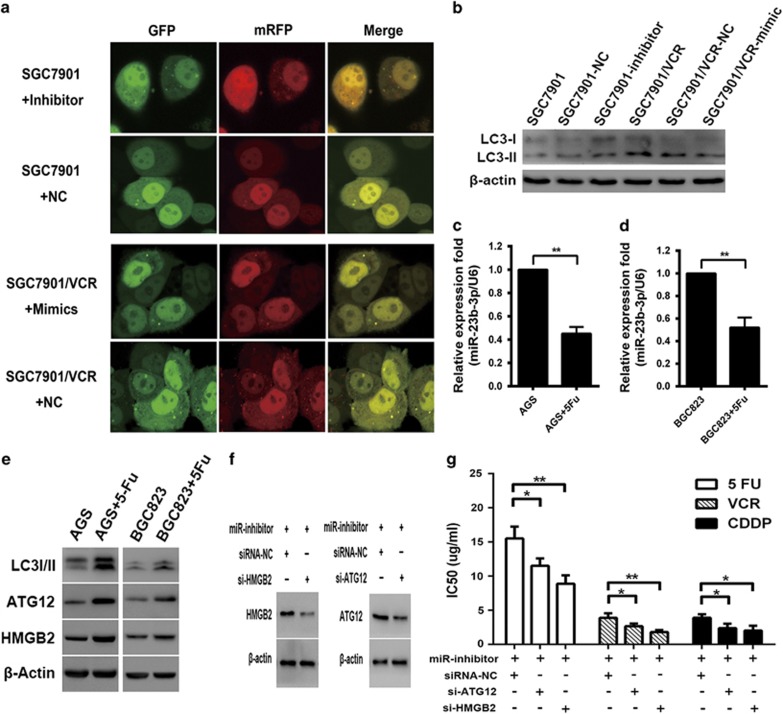
miR-23b-3p inhibits autophagy in chemoresistant cells by negatively regulating ATG12 and HMGB2. (**a**) GC cells stably expressing mRFP-GFP-LC3 were transfected with inhibitors, mimics or NC and then examined using CLSM. The white arrows indicate autophagosomes. (**b**) SGC7901/VCR and SGC7901 cells were transfected with mimics, inhibitors or NC. After 48 h, LC3-I/II proteins were detected using western blot analysis. (**c– e**) qRT-PCR and western blot analyses indicated that at 24 h after treating with low concentration of 5-Fu, miR-23b-3p expression in AGS and BGC823 cells was decreased, whereas the expressions of ATG12, HMGB2 and LC3-II were upregulated. (**f**) SGC7901 cells were cotransfected with inhibitors and siRNAs. After 48 h, ATG12 and HMGB2 were detected using western blot analysis. (**g**) Silencing of ATG12 or HMGB2 reverses the effect of miR-23b-3p knockdown on drug resistance. *P<0.05, **P<0.01

We next hypothesized that deregulation of miR-23b-3p may contribute to increased autophagy. To test this hypothesis, SGC7901/VCR or SGC7901 cells were transfected with miR-23b-3p mimics or inhibitors. At 48 h after transfection, the incidence of autophagy was confirmed. CLSM indicated that overexpression of miR-23b-3p in SGC7901/VCR cells significantly decreased the accumulation of autophagosomes. In contrast, inhibition of endogenous miR-23b-3p in SGC7901 cells showed the opposite effects (Figure 5a). We further observed that the overexpression of miR-23b-3p induced LC3 accumulation in SGC7901/VCR cells. In contrast, downregulation of miR-23b-3p led to increased expression of LC3II in SGC7901 cells (Figure 5b). In addition, qRT-PCR and western blot analysis indicated that at 24 h after treating with low concentration of 5-Fu, miR-23b-3p expression in AGS and BGC823 cells was decreased, whereas the expressions of ATG12, HMGB2 and LC3-II were upregulated (Figures 5c–e). We further cotransfected SGC7901 cells with miR-23b-3p inhibitors and siRNAs targeting ATG12 or HMGB2; the siRNAs targeting ATG12 and HMGB2 attenuated the effect of miR-23b-3p inhibitors (Figures 5f and g). These results confirm that miR-23b-3p regulates autophagy by targeting ATG12 and HMGB2.

### Restoration of miR-23b-3p increased the drug sensitivity of GC cells *in vivo*

**Figure 6:**
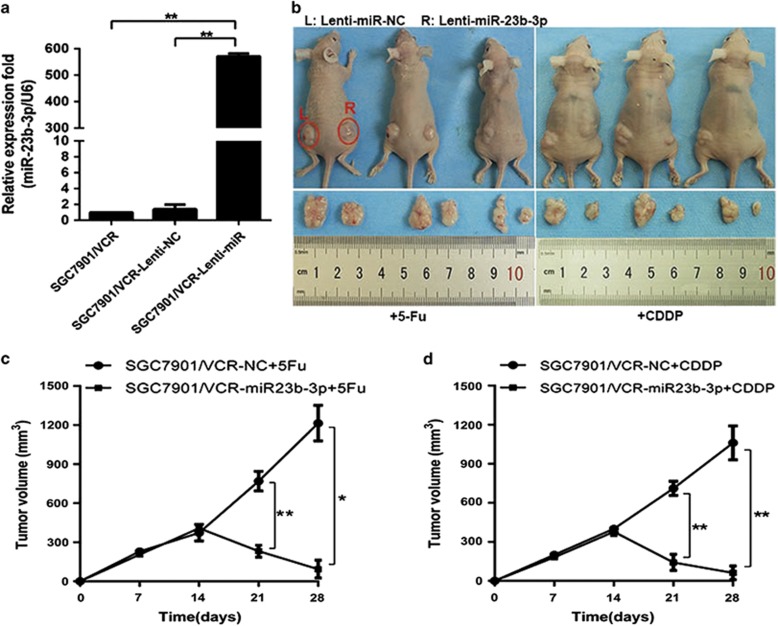
The restoration of miR-23b-3p reversed the drug resistance *in vivo*. (**a**) Expression of miR-23b-3p in SGC7901/VCR cells stably transfected with lenti-miR-NC or lenti-miR-23b-3p. (**b**) SGC7901/VCR-lenti-miR-NC- and SGC7901/VCR-lenti-miR-23b-3p-transfected cells were transplanted in the left and right side of the mice, respectively. (**c** and **d**) The tumor volumes were calculated as length × width^2^ and measured at the indicated time points. *P<0.05, **P<0.01

To investigate whether miR-23b-3p regulates the drug sensitivity of GC cells *in vivo*, we established SGC7901/VCR cells that stably express Lenti-NC or Lenti-miR-23b-3p. The efficiency of infection was confirmed using qRT-PCR (Figure 6a). We then transplanted SGC7901/VCR-Lenti-NC or SGC7901/VCR-Lenti-miR-23b-3p cells into nude mice. The volumes of the miR-23b-3p-transfected tumors were markedly decreased after chemotherapy, which indicated the reversion of drug resistance by ectopic miR-23b-3p expression (Figures 6b–d).

### miR-23b-3p expression is downregulated in chemoresistant patients and correlated with overall survival in patients with GC

**Figure 7:**
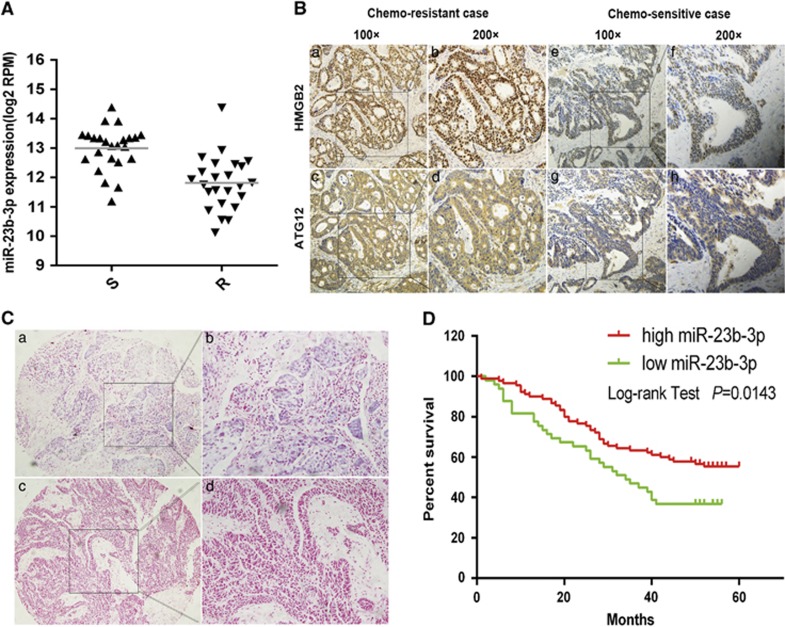
miR-23b-3p expression is inversely correlated with the expression of its target genes and correlates with overall survival in patients with gastric cancer. (**A** and **B**) Compared with chemosensitive patients (S), ATG12 and HMGB2 were markedly upregulated in chemoresistant patients (R), whereas the expression of miR-23b-3p was significantly decreased (*P*<0.01). (**C** and **D**) *In situ* hybridization and Kaplan–Meier curves were used to analyze the correlation between miR-23b-3p expression and overall survival

To verify the roles of miR-23b-3p in clinical samples, qRT-PCR and immunohistochemistry were used to detect miR-23b-3p and its targets, respectively. We found that the expression levels of miR-23b-3p were decreased in 24 chemoresistant patients, whereas the expression of ATG12 and HMGB2 were significantly upregulated in these patients compared with chemosensitive patients (Figures 7A and B). In addition, *in situ* hybridization was used to analyze the relation between miR-23b-3p expression and the clinicopathological parameters. These results showed that miR-23b-3p expression was statistically correlated with distant metastasis (*P*=0.005). However, there was no significant difference between miR-23b-3p expression and patient age, gender, differentiation, tumor depth, nodal metastasis and the TNM staging(Supplementary Table 3). Importantly, the patients in the low miR-23b-3p expression group had a significantly poorer prognosis than those in the high miR-23b-3p expression group (Figures 7C and D).

## Discussion

Primary and/or acquired resistance to chemotherapy remains a major obstacle to the clinical management of cancer types, resulting in relapse and metastasis in most malignant tumors. Although much research into the mechanisms of chemoresistance, such as increased drug efflux, mutation of target genes, inactivation of detoxification enzymes, dysfunction of pro-apoptotic proteins or enhancement of DNA repair activity,^21, 22^ has been reported, the mechanisms involved in cancer cell chemoresistance are still not clearly understood. Emerging evidence has demonstrated that miRNAs are involved in chemoresistance in many tumors, such as hepatocellular cancer, breast cancer and ovarian cancer.^23, 24, 25, 26^ However, there are relatively few studies on the MDR of GC.

According to the results of an miRNA array and high-throughput functional screening in our previous study,^14, 15^ we selected miR-23b-3p as a candidate gene for MDR in GC cells. Dysregulation of miR-23b-3p has been reported in many cancers, including colon cancer, prostate cancer, renal cancer and thymic lymphoma.^16, 17, 18, 27^ However, the roles of miR-23b-3p are not consistent in different cancers and are even conflicting within breast cancer,^28, 29^ and an association between miR-23b-3p and GC has not been previously reported. In the present study, the exact roles of miR-23b-3p in regulating MDR in GC cells were studied. The results showed that by targeting ATG12 and HMGB2, miR-23b-3p modulates the chemosensitivity of GC cells by mediating autophagy. To our knowledge, this report identifies miR-23b-3p as a key regulator of the chemosensitivity of GC for the first time.

miRNA usually has multiple target genes. To limit the range of candidate genes, we selected the target genes that were positive in all four prediction tools. The 10 candidate genes that were listed are as follows: *AUH*, *ATG12*, *HMGB2*, *NACC2*, *NDFIP2*, *RAB11FIP2*, *UBE2D1*, *WBP2*, *ZBTB34* and *ZNF238*. Among these genes, we first selected drug resistance-related genes. Compared with the other genes, *ATG12* and *HMGB2* were more related to chemoresistance of cancer. ATG12 is an important factor in autophagic vacuole formation.^30, 31^ Recent studies have reported that ATG12 is a novel determinant of chemoresistance or radioresistance.^19, 32^ Consistent with these reports, our results show that the expression of ATG12 was increased in MDR cells of GC. We also found that miR-23b-3p reduced ATG12 expression at both the mRNA and protein levels. Further knockdown of the expression of ATG12 by siRNA increased the sensitivity of MDR cells to chemotherapeutic agents, which suggests that ATG12 is associated with chemosensitivity in GC. HMGB2 is a member of the HMGB protein family, which comprises ubiquitous, abundant nonhistone nuclear proteins with diverse functions in the cell.^33^ The HMGB family consists of HMGB1, HMGB2, HMGB3 and HMGB4. Overexpression of HMGB1 has been observed in several human cancers, such as breast cancer and colon cancer.^34, 35^ Importantly, HMGB1 contributes to chemoresistance in many types of cancer by activating autophagy.^36, 37^ HMGB2 is highly homologous to HMGB1, and it may have similar effects with regard to cancer development. However, compared with HMGB1, relatively little is known regarding the biological function of HMGB2. Recently, it was reported that HMGB2 is overexpressed and promotes chemoresistance in glioblastoma and HCC.^38, 39^ In the present study, we found that the expression of HMGB2 was significantly higher in MDR GC cells than in the parental cells and that knockdown of HMGB2 significantly reversed MDR in GC. Similarly to ATG12, miR-23b-3p regulated HMGB2 by targeting its 3′-UTR. Thus, these results suggest that overexpression of HMGB2 promoted drug resistance in GC.

Emerging evidence indicates that autophagy is increased in several human cancers and contributes to chemoresistance.^37, 40^ ATG12 and HMGB2 were both overexpressed in MDR GC cells, which suggest that autophagy may be involved in MDR. To test this hypothesis, we detected the autophagic flux in our cell model. Consistent with the previous reports described above, our results indicated that MDR cells exhibited increased autophagy, which functions as a mechanism of chemoresistance. Reducing the expression of ATG12 or HMGB2 by administration of siRNA or CQ to MDR cells significantly decreased the level of autophagy, accompanied by increased sensitivity to drugs. Our data suggest that autophagy in MDR GC cells may be a survival mechanism that promotes chemoresistance and that inhibition of autophagy by interfering with ATG12 or HMGB2 has the potential to improve chemotherapeutic regimes.

Increasing research has revealed that miRNAs have an important role in regulating autophagy,^41^ including the induction or inhibition of autophagy. For example, forced expression of miR-155 increases autophagic activity in human nasopharyngeal cancer and cervical cancer cells;^42^ however, overexpression of miR-101 inhibits autophagy and enhances chemosensitivity both in HCC and osteosarcoma cells.^43, 44^ Therefore, different miRNAs may have different roles in regulating autophagy. Whether miR-23b-3p can regulate autophagy in GC chemoresistance is thus an important question. We modified the expression of miR-23b-3p by transfecting GC cells with miR-23b-3p mimics or inhibitors and found that upregulation of miR-23b-3p significantly inhibited autophagy in MDR cells. In contrast, downregulation of miR-23b-3p increased autophagy in the parental cells. However, whether these effects of miR-23b-3p are mediated by ATG12 and HMGB2 was still unknown. We cotransfected SGC7901 cells with miR-23b-3p inhibitors and siRNAs targeting ATG12 and HMGB2 and found that downregulation of ATG12 or HMGB2 by siRNAs reversed the effect of the miR-23b-3p inhibitor on autophagy. Thus, we have confirmed that miR-23b-3p inhibits autophagy by targeting ATG12 and HMGB2 in MDR GC cells, which suggests that miR-23b-3p may be a novel potential target for the treatment of GC.

Our study also showed that ATG12 was decreased at the protein level when HMGB2 was downregulated;however, ATG12 did not affect the expression level of HMGB2, possibly because of the transcription factor activity of HMGB2, which may regulate a wide range of molecules including ATG12. In addition, HMGB1 regulates autophagy in many cancers by stabilizing the HMGB1/Beclin1 complex.^36^ Because it is highly homologous to HMGB1, HMGB2 may have a similar role in regulating autophagy; however, this speculation must be verified in the further study. Collectively, our data reveal the direct and indirect regulation of autophagy by miR-23b-3p. First, overexpression of miR-23b-3p decreases ATG12 and HMGB2 expression, and, second, inhibition of HMGB2 by miR-23b-3p suppresses the expression of ATG12 or other autophagy-related molecules. We further validated the function of miR-23b-3p in MDR in a xenograft tumor model and clinical GC specimens, showing that this miRNA also regulated drug resistance *in vivo*. Meanwhile, the data of tissue microarrays showed that miR-23b-3p did not correlate with any clinicopathological parameters except distant metastasis. Interestingly, several research have been confirmed that miR-23b regulated metastasis of many cancers including breast cancer,^29^ colon cancer,^16^ bladder cancer^45^ and prostate cancer^27^ which suggested that miR-23b-3p might be involved in metastasis of GC. In addition, Kaplan–Meier analysis revealed that the prognosis of GC patients was significantly related to the miR-23b-3p expression level. This result indicated that miR-23b-3p might serve as an MDR-specific miRNA in GC and could be used as the predictor for overall survival.

We have confirmed that miR-23b-3p was downregulated in chemoresistant cells and tissues of GC. To further investigate whether chemotherapy drugs influenced on the expression of miR-23b-3p, we performed 5-Fu that was widely used in clinical setting to treat two typical gastric adenocarcinoma cells AGS and BGC823. The results indicated that 5-Fu decreased the expression of miR-23b-3p. However, much more work would be performed to elucidate the exact effects of chemotherapeutics on miR-23b-3p. In addition, several studies have reported that DNA methylation and transcription factors such as c-Myc^46, 47, 48^ contribute to the silencing of miR-23b-3p in different types of cancer; however, the underlying mechanism of dysregulation of miR-23b-3p in GC chemoresistance remains unclear. Thus, further studies should be performed to address the above questions.

In summary, our data reveal that miR-23b-3p is a novel miRNA that regulates MDR in GC. Overexpression of miR-23b-3p inhibits autophagy in MDR cells by targeting ATG12 and HMGB2 and increases the sensitivity of MDR cells to chemotherapeutics. Although further studies are needed to fully elucidate the definite regulatory mechanisms of miR-23b-3p, this work suggests the novel miR-23b-3p/ATG12/HMGB2/autophagy-regulatory loop characterized here provides new insight into the mechanisms underlying drug resistance, and restoration of miR-23b-3p expression may be a potential therapeutic strategy for the treatment of MDR in GC.

## Materials and Methods

### Cell culture

The human gastric adenocarcinoma cell line SGC7901 (obtained from the Academy of Military Medical Science, Beijing, China), SGC7901/VCR (established and maintained in our laboratory), AGS and BGC823 were cultured in RPMI-1640 or DMEM medium (HyClone, Logan, UT, USA) supplemented with 10% fetal calf serum (Gibco, Carlsbad, CA, USA) and 100 *μ*g/ml streptomycin sulfate at 37 °C in a humidified air atmosphere containing 5% CO_2_. To maintain the MDR phenotype, VCR was added to the culture medium of SGC7901/VCR cells at a final concentration of 1 *μ*g/ml.

### RNA extraction and quantitative real-time PCR

Total RNA was extracted from cells or tissues using TRIzol reagent (Invitrogen, Carlsbad, CA, USA). To detect the expression of miR-23b-3p, qRT-PCR was performed using an All-in-One miRNA Detection Kit (GeneCopoeia, Rockville, MD, USA) according to the manufacturer's instructions. The specific qRT-PCR primers for miR-23b-3p and U6 were purchased from GeneCopoeia. A SYBR Green PCR Kit (Takara Bio Inc., Otsu, Japan) was used to determine the expression level of ATG12 and HMGB2 mRNA. Glyceraldehyde 3-phosphate dehydrogenase was used as an endogenous control for RNA normalization. Each sample was analyzed in triplicate.

### Oligonucleotide construction and lentivirus production

The miR-23b-3p mimics, inhibitors and negative control construct were all purchased from RiboBio Co. Ltd (Guangzhou, Guangdong, China). siRNAs targeting ATG12 and HMGB2 were purchased from GenePharma (Shanghai, China). Oligonucleotide transfection was performed using Lipofectamine RNAiMAX Transfection Reagent (Invitrogen) in accordance with the manufacturer's procedures. Stable transfectants overexpressing miR-23b-3p or the negative control construct were generated using the pEZX-MR03 lentiviral transfer vector (GeneCopoeia). The lentiviral vector control only expressed green fluorescent protein.

### *In vitro* and *in vivo* drug-sensitivity assay

SGC7901 and SGC7901/VCR cells were plated in six-well plates (3 × 10^5^cells/well) in an antibiotic-free medium and transfected with oligonucleotides using Lipofectamine RNAiMAX transfection reagent (Invitrogen) according to the manufacturer's protocol. At 48 h after transfection, 5 × 10^3^ cells were seeded into each well of a 96-well plate, and the medium containing the anticancer drugs VCR, 5-fluorouracil (5-FU) and cisplatin (CDDP) at different concentrations was added to each well. After the plates were incubated for 48 h, the MTT (Sigma, St Louis, MO, USA) assay was performed. The concentration at which each drug produced IC_50_ was then calculated. For *in vivo* experiments, ~1.0 × 10^7^ SGC7901/VCR cells stably transfected with lenti-miR-23b-3p or lenti-NC were subcutaneously injected into both flanks of nude mice. Two weeks later, the mice were intraperitoneally injected with PBS containing 5-Fu or CDDP (10 mg/kg) once per week. The mice were humanely killed on day 28, and the tumors were measured and photographed.

### Vector construction and luciferase reporter assay

Luciferase assays were performed as described previously.^12^ Briefly, SGC7901/VCR cells were cotransfected with plasmids expressing wild-type Luc-ATG12, mutant Luc-ATG12, wild-type Luc-HMGB2 or mutant Luc-HMGB2 3′-UTR (GeneCopoeia) in addition to the miR-23b-3p mimics (at a final concentration of 50 nmol/l). At 24 h after transfection, the firefly and Renilla luciferase activities were quantified using a dual luciferase assay kit (GeneCopoeia).

### Electron microscopy

Cells were fixed in 2.5% glutaraldehyde containing 0.1 mol/l sodium cacodylate for 2 h or more, incubated in 1% osmium tetroxide, dehydrated with an increasing concentration gradient of ethanol and acetone, embedded in Araldite, cut into 50-nm sections and stained with 3% uranyl acetate and lead citrate. Images were generated using a Philips CM-120 TEM (Amsterdam, Netherlands).

### Stably expressing mRFP-GFP-LC3 and CLSM

The adenovirus vector containing the mRFP-GFP-LC3 reporter was purchased from Hanbio (Shanghai, China). Stable EGFP-positive cells were isolated using flow cytometry. The CLSM analysis was performed as previously described.^49^ Briefly, the cells were incubated with primary antibodies recognizing ATG12 and HMGB2 (rabbit, Abcam, Cambridge, UK) overnight at 4 °C, and then fixed and incubated with AffiniPure donkey anti-rabbit IgG (1 : 200; red fluorescence, Jackson ImmunoResearch Laboratories Inc., Baltimore, MD, USA) as the secondary antibody for 1 h at room temperature. DAPI (Beyotime, Shanghai, China) was applied for 5 min to stain the nuclei. The cells were then analyzed using confocal microscopy (FV10i, Olympus, Tokyo, Japan).

### Western blot analysis

Protein samples (cells or tissues) were solubilized in RIPA (Beyotime) containing protease inhibitors (Roche, Switzerland), and western blot analysis was performed as previously described.^50^ Primary antibodies against P62 (1 : 500, Santa Cruz), ATG12 (1 : 1000, Abcam), LC3I/II (1 : 1000, Cell Signaling Technology (CST)) and HMGB2 (1 : 1000, CST) were purchased from Santa Cruz, Abcam orCST. An antibody against β-actin (Sigma) was used as an internal control.

### Immunohistochemistry

Forty-eight GC patients (24 chemoresistant cases and 24 chemosensitive cases) who received neoadjuvant chemotherapy before surgery between 2012 and 2014 were obtained from Xijing hospital. The protocol was approved by the human ethics committee at the Fourth Military Medical University. Patients offering samples for the study signed informed consent forms. The immunohistochemical staining of ATG12 and HMGB2 was performed as previously described.^49^ The immunostaining results were scored as the percentage of cells staining positive as follows: 0 for <1% of cells, 1 for 1–25% of cells, 2 for 26–50% of cells, 3 for 51–75% of cells and 4 for >75% of cells. Staining intensity was graded as follows: 0, no staining; 1, weak staining; 2, moderate staining; and 3, strong staining. The histological score (H-score) of ATG12 and HMGB2 for each section was computed by the following formula: H-score=ratio score × intensity score. A total score of 0–12 was graded as negative (−, score 0–1), weak (+, score 2–4), moderate (++, score 5–8) or strong (+++, score 9–12) for further nonparametric testing.

### Tissue microarrays and *in situ* hybridization

Tissue microarrays which included 140 cases of gastric malignant tissues with detailed clinicopathological and follow-up information were purchased from shBiochip (Shanghai, China). *In situ* hybridization was performed using a miR-23b-3p probe from GeneCopoeia. The probe was detected using digoxigenin antibody (Abcam), LSAB2 System-HRP (Dako Denmark A/S, Glostrup, Denmark) and liquid DAB+ Substrate Chromogen System (Dako) according to the manufacturer's instructions. The scoring of hybridization was the same as mentioned. Scoring with ‘−' and ‘+' was regarded as lower miR-23b-3p expression, whereas ‘++' and ‘+++' represented higher expression of miR-23b-3p. The results of immunostaining and hybridization were independently scored by two pathologists in a blind manner.

### Statistical analysis

Student's *t*-test (two-tailed) or a one-way analysis of variance was employed to analyze the *in vitro* and *in vivo* data. The Kruskal–Wallis H-test and the Mann–Whitney *U*-test were used to analyze the relationships between miR-23b-3p expression levels and clinicopathological factors. Survival curve was estimated using the Kaplan–Meier method, and the differences in survival distributions were evaluated by the Log–rank test. *P*-values <0.05 were considered statistically significant. All statistical analyses were performed using the SPSS version 17.0 software package (SPSS Inc., Chicago, IL, USA).

## Abbreviations

miRNA: microRNA
3′-UTR: 3-untranslated regions
MDR: multidrug resistance
ATG12: autophagy-related gene 12
HMGB2: high-mobility group box 2
CLSM: confocal laser scanning microscopy
TEM: transmission electron microscopy
