# Supplementary figures and images for: miR-23b-3p regulates the chemoresistance of gastric cancer cells by targeting ATG12 and HMGB2
Source: Cell Death Dis. 2015 May 21;6(5):e1766–. doi: 10.1038/cddis.2015.123 (PMC4669702; doi:10.1038/cddis.2015.123)

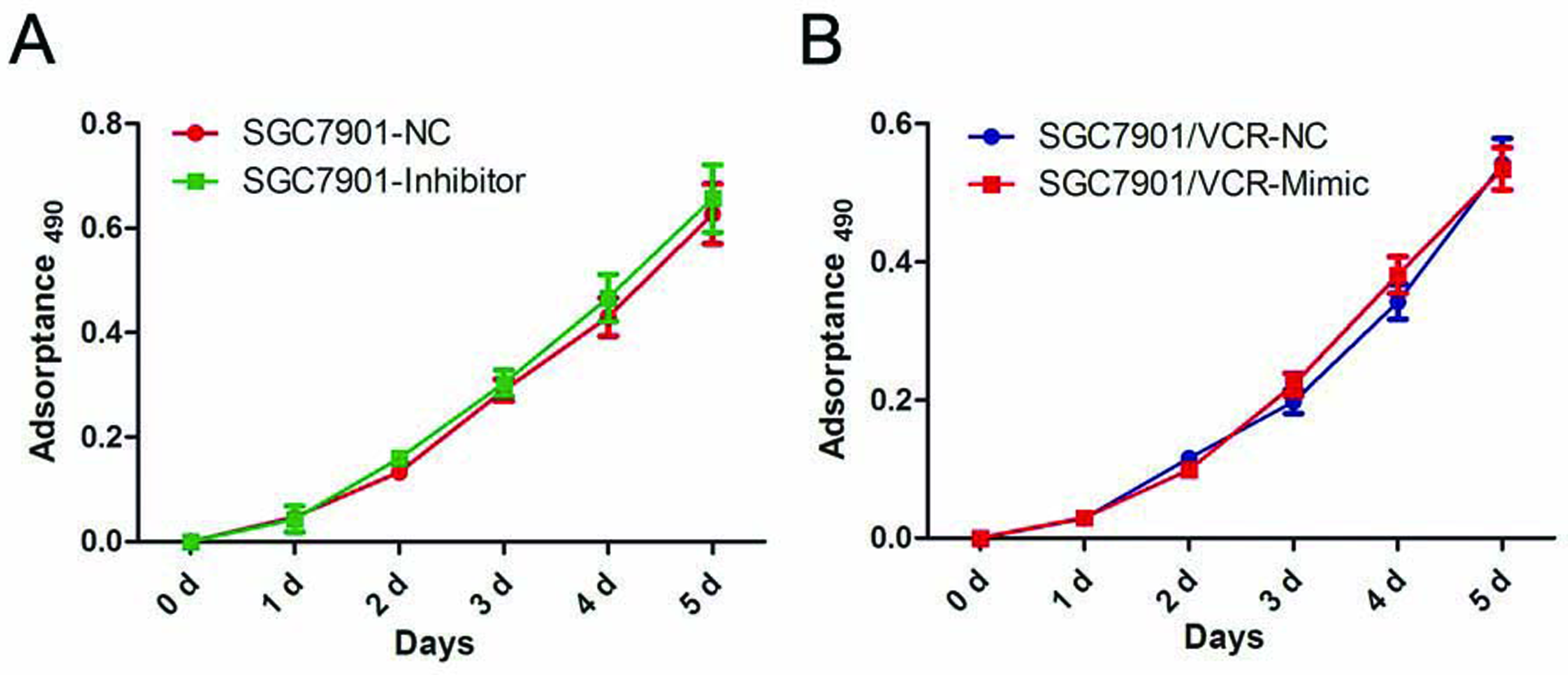

Supplement: Supplementary Figures [file cddis2015123x1.tif]
