# Supplementary material for: miR-23b-3p regulates the chemoresistance of gastric cancer cells by targeting ATG12 and HMGB2
Source: Cell Death Dis. 2015 May 21;6(5):e1766–. doi: 10.1038/cddis.2015.123 (PMC4669702; doi:10.1038/cddis.2015.123)
Supplement: Supplementary Table 1 [file cddis2015123x2.doc]

Table 1 List of genes and primer sequences.

| Gene | Primer* | (5´-3´) |
| --- | --- | --- |
| HMGB2 | F | GAGCAGTCAGCCAAAGATAAACAA |
|  | R | AGCACACACACACATTCCACAC |
| ATG12 | F | TGACCTGCTGGCTGAATACCT |
|  | R | GATGTGAAACCAAAACGCCTAAC |

*Primer = Forward (F), Reverse (R).
