# Supplementary material for: miR-23b-3p regulates the chemoresistance of gastric cancer cells by targeting ATG12 and HMGB2
Source: Cell Death Dis. 2015 May 21;6(5):e1766–. doi: 10.1038/cddis.2015.123 (PMC4669702; doi:10.1038/cddis.2015.123)
Supplement: Supplementary Table 2 [file cddis2015123x3.doc]

Table 2. siRNA and primer sequences

**Gene sense（5'-3'） antisense（5'-3'）**

| HMGB2-Homo-297 | CCGUCAAUUUCGCGGAAUUTT | AAUUCCGCGAAAUUGACGGTT |
| --- | --- | --- |
| HMGB2-Homo-358 | GGAGAAGUCGAAGUUUGAATT | UUCAAACUUCGACUUCUCCTT |
| HMGB2-Homo-605 | GCCAAAGAUAAACAACCAUTT | AUGGUUGUUUAUCUUUGGCTT |

| ATG12-Homo-321 | GUUGCAGCUUCCUACUUCATT | UGAAGUAGGAAGCUGCAACTT |
| --- | --- | --- |
| ATG12-Homo-1013 | CUGGCUGAAUACCUCAAAUTT | AUUUGAGGUAUUCAGCCAGTT |
| ATG12-Homo-866 | GAGACUAAGACUGUAUAAATT | UUUAUACAGUCUUAGUCUCTT |
