# Supplementary material for: miR-23b-3p regulates the chemoresistance of gastric cancer cells by targeting ATG12 and HMGB2
Source: Cell Death Dis. 2015 May 21;6(5):e1766–. doi: 10.1038/cddis.2015.123 (PMC4669702; doi:10.1038/cddis.2015.123)
Supplement: Supplementary Table 3 [file cddis2015123x4.doc]

Table 3 Association of miR-23b-3p expression with clinicopathologic characteristics

| Variables | No. of cases | Expression of miR-23b-3p  Low (%) High (%) | *p-value^*^* |
| --- | --- | --- | --- |
| Age (years) |  |  | 0.388 |
| ≥ 50 | 114 | 38(33.3) 76(66.7) |  |
| < 50 | 26 | 11(42.3) 15(57.7) |  |
| Gender |  |  | 0.161 |
| Male | 77 | 23(29.9) 54(70.1) |  |
| Female | 63 | 26(41.3) 37(58.7) |  |
| Degree of differentiation |  |  | 0.926 |
| Well and moderately differentiated | 121 | 42(34.7) 79(65.3) |  |
| Poorly differentiated | 19 | 7(36.8) 12(63.2) |  |
| T classification |  |  | 0.263 |
| T1 | 10 | 5(10.2) 5(5.5) |  |
| T2 | 42 | 10(20.4) 32(35.2) |  |
| T3 | 81 | 30(61.2) 51(56) |  |
| T4 | 7 | 4(8.2) 3(3.3) |  |
| N classification |  |  | 0.733 |
| N0 | 83 | 30(61.2) 53(58.2) |  |
| N≥1 | 57 | 19(38.8) 38(41.8) |  |
| M classification |  |  | 0.005 |
| M0 | 117 | 35(71.4) 82(90.1) |  |
| M1 | 23 | 14(28.6) 9(9.9) |  |
| TNM stage |  |  | 0.297 |
| Stage I/II | 99 | 30(30.3) 69(69.7) |  |
| Stage III/IV | 41 | 19(46.3) 22(53.7) |  |

*The Kruskal–Wallis H-test and the Mann–Whitney U test were used to analyze the relationship between miR-23b-3p expression and clinicopathologic characteristics
