# Supplementary material for: miR-23b-3p regulates the chemoresistance of gastric cancer cells by targeting ATG12 and HMGB2
Source: Cell Death Dis. 2015 May 21;6(5):e1766–. doi: 10.1038/cddis.2015.123 (PMC4669702; doi:10.1038/cddis.2015.123)
Supplement: Supplementary Material [file cddis2015123x5.doc]

**Supplemental Materials**

**Figure 1.** The effects of miR-23b-3p on the cell growth in gastric cancer cells

**Table 1.** List of genes and primer sequences

**Table 2.** siRNA and primer sequences

**Table 3.** Association of miR-23b-3p expression with clinicopathologic characteristics
